# Amorphous calcium phosphate nanoparticles could function as a novel cancer therapeutic agent by employing a suitable targeted drug delivery platform

**DOI:** 10.1186/1556-276X-8-449

**Published:** 2013-10-30

**Authors:** Milad Pourbaghi-Masouleh, Vahid Hosseini

**Affiliations:** 1 Nanotechnology and Advanced Materials Department, Materials and Energy Research Center, Karaj, P.O. Box: 31787/316, Iran; 2 Department of Health Science and Technology, Laboratory of Applied Mechanobiology, ETH, Zürich 8093, Switzerland

**Keywords:** Amorphous calcium phosphate nanoparticle, Apoptosis, Cancer, Calcium, Endosomal escape, Targeting ligand

## Abstract

Employment of nanovehicular system for delivering apoptogenic agent to cancer cells for inducing apoptosis has widely been investigated. Loading efficacy and controlled release of the agents are of the inseparable obstacles that hamper the efforts in reaching an efficacious targeted cancer therapy method. When the carrier itself is apoptogenic, then there is no need to load the carrier with apoptogenic agent and just delivering of the particle to the specific location matters. Hence, we hypothesize that amorphous calcium phosphate nanoparticle (ACPN) is a potent candidate for apoptosis induction, although encapsulation in liposome shell, and surface decoration with targeting ligand (TL), and cell-penetrating peptide (CPP) plays a pivotal role in the employment of this agent. It is well understood that elevation in cytosolic Ca^2+^ ([Ca^2+^]_c_) would result in the induction of apoptosis. ACPN has the potential to cause imbalance in this medium by elevating [Ca^2+^]_c_. Owning to the fact that the nanoparticles should be delivered into cytosol, it is necessary to trap them in a liposomal shell for evading endocytosis. It was demonstrated that employment of the trans-activator of transcription (TAT) as CPP eminently enhances the efficacy of endosomal escape; therefore, the platform is designed in a way that TAT is positioned on the surface of the liposome. Due to the fact that the apoptosis should be induced in sole cancer cells, Folate as TL is also attached on the surface of the liposome. This hypothesis heralds the new generation of chemotherapeutic agents and platforms which could have less side effect than the most common ones, in addition to other advantages they have.

## Background

Many chemotherapeutic agents with different mechanisms of action have been developed up to now. Apoptosis induction is one of the mechanisms which has attracted researchers' attention for fighting against cancer [1]. Doxorubicin [2], daunorubicin [3], idarubicin [4], bleomycin [5], mitomycin C [6], cisplatin [7], plicamycin [8], and carmustine [9] are of the well-known apoptogenic agents; although in order to serve them in targeted drug delivery system, appropriate drug carriers should be employed. Such carriers are aimed to facilitate drug delivery procedure and avoid problems like bioavailability and normal tissue toxicity. In this regard, the issues such as loading efficacy and controlled release of the agents are of the inseparable obstacles that researchers are confronted with up to now. The development of an agent that possesses the favorable drug carrier characteristics and acts as an apoptogenic agent by itself could be a promising method for coping with the mentioned obstacles.

Calcium phosphate minerals are mostly known as bone substitutive materials due to their outstanding biocompatibility [10]. Employing nanotechnology has led to develop these biomaterials in nanoscale range, although some of the studies reported the cytotoxic effect of hydroxyapatite (one of calcium phosphate crystalline phases) nanoparticles (HANs) on bone and cartilage cells through apoptosis induction [11-16]. This adverse effect was also observed in other cell lines such as macrophage, granulose, epithelial, and muscle [17-20]. Interestingly, it was demonstrated that HANs also could have toxic effect on cancer cells through triggering the apoptosis, which leads to cell death and inhibits proliferation [12,21-28]. Based on the abovementioned facts, it could be suggested that HAN has the potential to serve as an apoptogenic agent.

Always, there are associate risks and adverse effects of administrated chemicals, drugs, and medicine via nanocarriers such as calcium phosphate nanoparticles (CPNs). In this study, it is aimed to reduce such risks by employing CPN as an anticancer agent, not as a drug carrier. This hypothesis is not in contrast with using CPNs as drug delivery vehicles and it can be used for such purposes such as gene delivery, but here, the potency of amorphous calcium phosphate nanoparticles (ACPNs) for cancer therapy is highlighted. As long as this hypothesis matters, two issues are brought up: (i) whether only HAN induces this effect in cells or other CPNs possess this potential and (ii) the steps toward development of a favorable platform in order to be utilized in cancer therapy. Therefore, through the presented hypothesis, we suggest that ACPN could serve as an apoptogenic agent in cancer treatment by employing a suitable targeted drug delivery platform.

## The hypothesis

**Figure 1:**
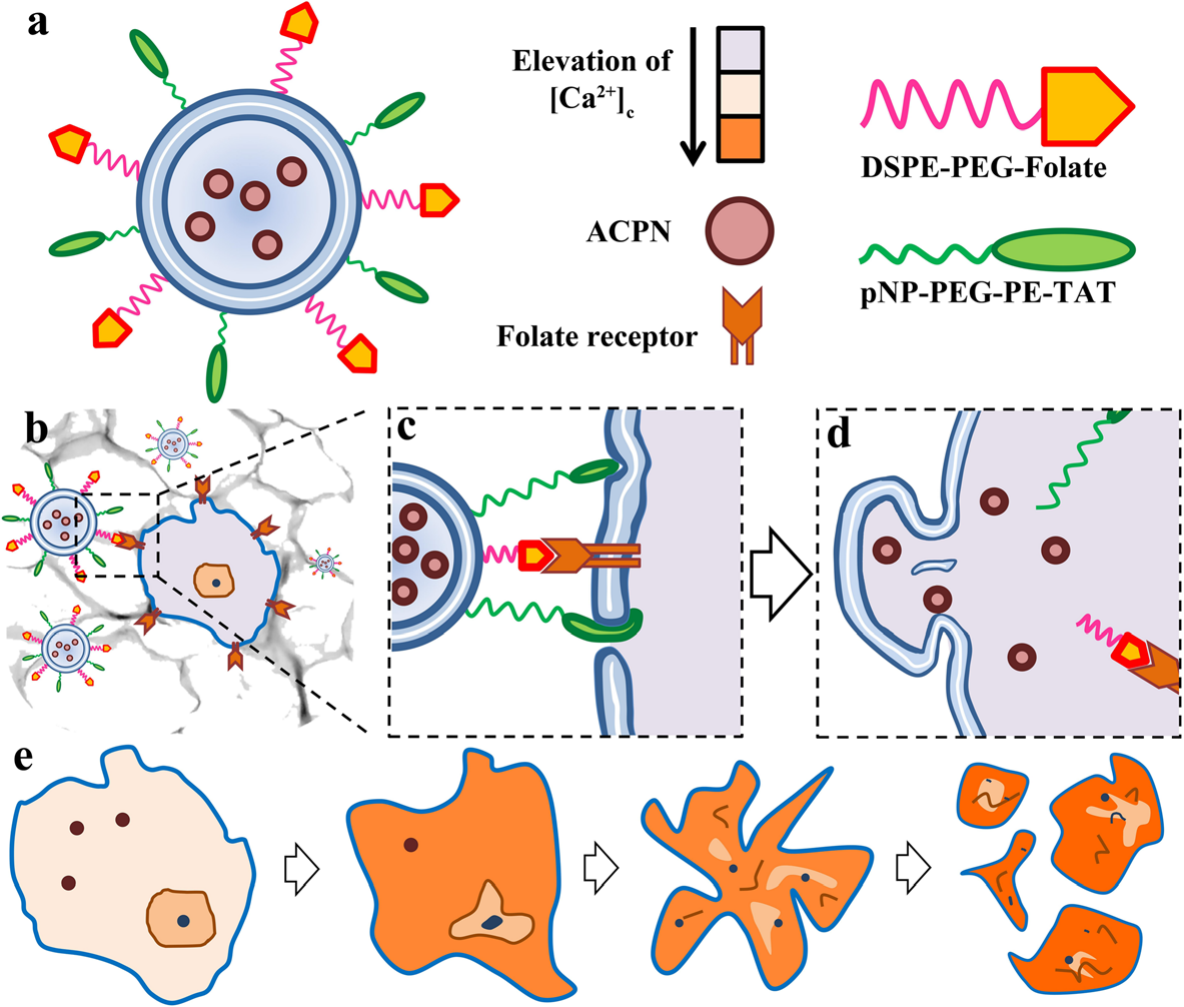
**Schematic diagram of the designed platform and its mechanism of action. (a)** the structure of the platform, **(b)** targeting on cancer cell, **(c)** penetration of CPP in liposomal membrane, **(d)** intracellular release of ACPNs, **(e)** explosion of cancer cell into a cascade of apoptotic body.

Considering the various obstacles in employing the chemotherapeutic agents and the problems in their delivery, we suggest ACPN as a chemotherapeutic agent in order to be served in cancer treatment. In order to employ ACPN for this purpose, it should be loaded in a liposomal shell decorated with TLs and CPPs. As it can be seen in Figure 1a, a designed platform comprises the ACPNs, which are trapped in a liposomal shell, and folate as TL and TAT as CPP which are both positioned on the surface of the liposome.

All the studies which have been done up to now, in order to study the toxicity of CPN, are focused on HANs. The other phases of calcium phosphate minerals have not been investigated concerning their nanotoxicity. It should be noticed that the particle could not be toxic by itself. However, the products of particle dissolution and their effect on cellular mechanism lead to the induced cytotoxicity. Considering the HANs dissolution, Ca^2+^, PO_4_^3−^, and OH^−^ are the ions (products) which leach out into the biological medium surrounding the particle. Hence, we hypothesize that ACPN could be more capable of inducing the apoptosis in comparison to HAN. In fact, the amorphous phase of calcium phosphate is far more degradable than the crystalline phases of calcium phosphate minerals such as hydroxyapatite. It is worthy of mention that the apoptosis could be triggered while [Ca^2+^]_c_ augments. This fact suggests that the ACPN should be intracellularly dissolved by cytosol, so it necessitates delivering the cargo to cytosol through an endosomal escape pathway and the best condition happens when the endocytosis does not occur. Therefore, the ACPN should be trapped in a liposomal capsule in order to deliver the nanoparticles through endosomal escape pathway. Although employment of liposome could lead to endosomal escape, it is demonstrated that presence of TAT peptides on the surface of the platform significantly enhances the efficacy of intracellular delivery.

Effective elimination of foreign materials from the circulation by the reticuloendothelial system (RES) is counted as one of the major problems of drug delivery system [29]. While nanoparticles have solved many problems in drug delivery, elimination by the RES has remained an obstacle up to now. Nanoparticle size and surface charge are the two major properties strongly influencing the elimination by this system [30,31]. Although the main established mechanisms for clearance of calcium phosphates are phagocytosis and acidification [32], the RES is also capable of eliminating them [33]. Since CPNs are advantageous for the delivery of therapeutics [34], for improving the efficiency of therapy, evading RES seems necessary for nanoparticles. In this regard, the prepared platform should also be superficially decorated with folate in order to enhance the cell-specific delivery of the ACPN.

## Testing the hypothesis

Contrary to the previous studies, we believe that ACPN could be more efficient in inducing apoptosis in cells, when they are delivered into the cytosol. This hypothesis is based on this fact that the elevation in [Ca^2+^]_c_ could lead to apoptosis induction through both caspase-dependent and caspase-independent pathways [35,36]. According to far higher dissolution rate of ACPN in comparison to HAN [37], more calcium concentration can be provided through the dissolution of ACPN in the cytosol. According to the mentioned studies, the HAN was just mediated with the cells. Accordingly, it is reported that nanoparticles escaping from endosomes are located in the cytosol and their dissolution resulted in the elevation of [Ca^2+^]_c_[17], while no endosomal escape platform was provided. In the case of employing ACPN, higher elevation of [Ca^2+^]_c_ is rapidly provided and the cell lacks the appropriate amount of time to pump out the extra intracellular calcium [38]. Hence, the delivery platform is designed in a way that delivers the ACPN into the cytosol utilizing a liposomal capsule [39]. The presence of this capsule results in the endosomal escape of the trapped ACPNs and the nanoparticles could be released into the cytosol; although, like other experiments, efficacy matters. In order to enhance the efficacy of endosomal escape, the surface of the liposome should be decorated with TAT peptides which dramatically raise the rate of intracellular delivery [40]. TAT peptide molecules should be attached on the liposome surface via pNP-PEG-PE spacer [41]. Folate is often used as a targeting ligand which has high specificity and affinity for cell surface to the folate receptor, which is over-expressed in some cancer cells including the breast, lung, kidney, ovary, and brain, among others [42]. Folate could be attached on the liposome surface utilizing DSPE-PEG-FOL [43]. The presence of polyethylene glycol (PEG) could provide a protective shield which leads to the avoidance of immune detection [44].

The hypothesized delivery platform has the potential to target cancer cells through binding the targeting ligands to Folate receptors. While the cell finds the specific cells, TAT peptide can generate saddle-splay membrane curvature and enter through an induced pore [45]; thereafter, liposome fusion happens, and consequently, the ACPNs enter the cytosol. As is mentioned before, dissolution of each ACPN results in [Ca^2+^]_c_ elevation which eventually leads to cell death through the triggering of apoptosis Figure 1b,c,d,e.

**Figure 2:**
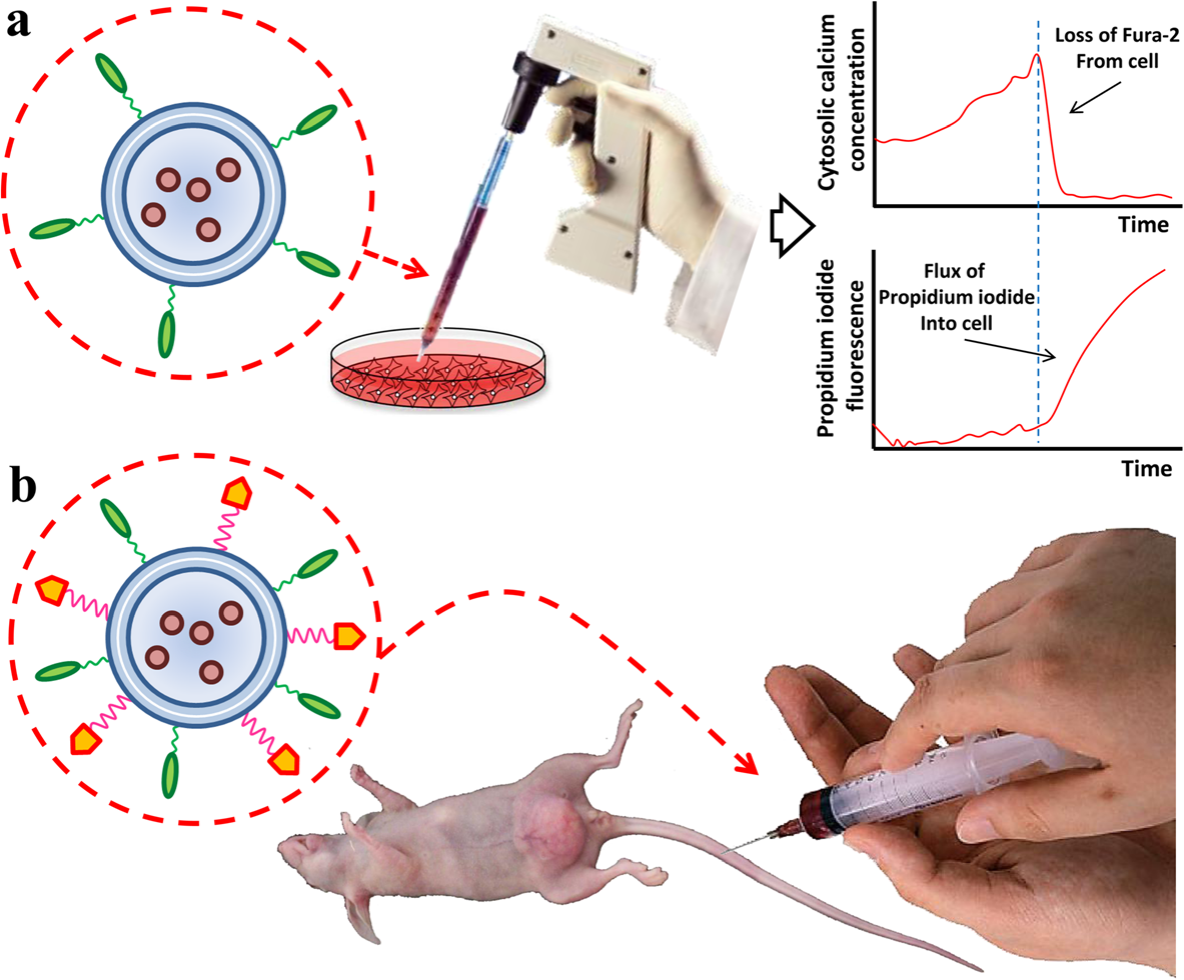
**Experimentation with the developed platform: (a) *in vitro *** **study, (b) *in vivo *** **study.**

In order to find the appropriate dosage of ACPN for apoptosis induction, an *in vitro* experiment should be conducted. A type of cancer cell such as glioma cell is cultured. Since in this part of study, targeting is out of importance, the platforms are prepared in the absence of folate. ACPN-loaded platforms, without a targeting ligand, are added to the culture dish. Regarding the fact that elevation in [Ca^2+^]_c_ determines when the cell starts apoptosis, in this part of study, the point is to find the amount of [Ca^2+^]_c_ introduced by each ACPN. Hence, measurement of [Ca^2+^]_c_ could be performed by monitoring Fura-2 fluorescence of cancer cells adhered to the dish using a proper imaging system. Fura-2 is loaded into the cells by the proper amount of incubation time. In order to investigate the integrity of cell membrane, which is related to [Ca^2+^]_c_, Fura-2/propidium iodide assay is employed. Further details for both measurements are presented by Ewence et al. [20] (Figure 2a). Obtained data from this part of study shows appropriate dosage of ACPNs and efficient exposure time. These results are based on the type of cancer cell that has been experimented.

Due to the fact that this platform is decorated with folate as a targeting ligand, in order to investigate the efficiency of the method and tumor accumulation of ACPN, an *in vitro* experiment should be conducted. In this regard, the proper dosage of ACPN should be injected intravenously into a mouse bearing glioma xenograft, according to a predetermined schedule. Since the injection is intravenous and not intratumoral, the platform should be decorated by folate. The size of tumors is measured in different intervals. Moreover, the tissue of tumors should be observed by terminal deoxynucleotidyl transferase dUTP nick end labeling (TUNEL) assay in order to compare the amount of apoptotic cells (Figure 2b).

## Implications of the hypothesis

Utilization of chemotherapeutic agents has been common for cancer treatment up to now. For efficient employment of such chemotherapeutic agents, appropriate carriers should be employed. Many attempts have been made to overcome the obstacles that hinder drug delivery system by applying nanotechnology to the preparation of suitable carriers. Even though nanotoxicity has adverse effect on normal cells, such toxicity could be employed to kill abnormal cells. As it is well proven, both chemotrapeutics and nanoparticles have induced toxicity to normal cells. Reducing this risk is the biggest challenge for both systems. ACPNs exactly meet these conditions due to the fact that extracellularly released nanoparticles cleared through the RES, although the particles should be targeted by the suggested platform. Regarding the suggested platform, the RES could not hinder circulation. The employment of PEG on the surface of the liposome could result in a structure that prolongs circulation of the trapped drug, or in this study, ACPNs. Moreover, macrophages in the RES located in the liver and the spleen take up particles bound with serum proteins; therefore, surface modification by PEG reduces the opsonization of liposomes and reduces the clearance by the RES, leading to enhanced pharmacokinetic properties [46]. Evading the RES will result in the effective accumulation of ACPNs in the tumor by the enhanced permeability and retention (EPR) effect. This effect facilitates drug release within the target tissues. In this study, employment of folate as a targeting ligand also results in EPR elevation [47]. In the near future, probably lots of these platforms will be developed in order to avoid drug delivery obstacles, although this hypothesis is the first one of its kind.

Although bioaccumulation of ACPNs has not been studied in particular, the distribution of HANs in mouse organs was studied *via* intravenous administration. Accordingly, after 1 h of HANs circulation, the lung, liver, and spleen contained most concentration of the nanoparticles, which were sixfold higher than other organs. After 72 h, however, the amount of these nanoparticles decreased significantly in three organs, suggesting that the HANs can be metabolized or excreted through these organs. A gradual reduction in the concentration of HANs was also detected in other organs which suggests that considerable amount of nanoparticles have been metabolized or excreted. It is worthy of mention that this amount remained constant in the bone. Interestingly, it was reported that the concentration of calcium always increases with time in the excrement of mice. It can be obviously attributed to the macrophages in the spleen, lung, and liver, where HANs are captured in. The nanoparticles in macrophages can be metabolized by the common bile duct and finally excluded from the body *via* feces. Moreover, it was found that only very low concentration of calcium is detected in the urine, suggesting nanoparticles are not excreted from the body *via* the kidney [48].

The designed platform is actually for apoptosis induction in cancer cells, although further consideration is needed in order to find the critical dosage of ACPN which should be uptaken by specific cancer cells to provide the appropriate [Ca^2+^]_c_ elevation for triggering apoptosis and avoiding necrosis [49]. Selection of an appropriate ligand with suitable water solubility should also be investigated in order to enhance the cell-specific targeting [50]. There are also some issues on calcium-phosphate ratio in ACPN which affect the rate of dissolution in biological mediums [37]. Understanding this ratio could also influence the rate of apoptosis induction, so it needs to be considered. Regarding the induction of apoptosis by nanoparticles such as ACPNs, we propose ‘*Nanoptosis’* as a scientific name for this phenomenon. Consequently, the nanoparticles that could result in *Nanoptosis* are called *‘Nanoptogenics’.*

## Abbreviations

ACPN: Amorphous calcium phosphate nanoparticle; HAN: Hydroxyapatite nanoparticle; CPN: Calcium phosphate nanoparticle; TL: Targeting ligand; CPP: Cell-penetrating peptide; [Ca2+]c: cytosolic Ca^2+^; TAT: Trans-activator of transcription.

## Competing interests

The authors declare that they have no competing interests.

## Authors' contributions

MPM brainstormed and developed the idea and drafted the manuscript. VH contributed in development of the idea and drafted the manuscript. Both authors read and approved the final manuscript.
